# Adsorption characteristics of modified bentonites for purification of anthocyanin from saffron tepal

**DOI:** 10.1002/fsn3.4388

**Published:** 2024-09-25

**Authors:** Hasan Oliaei Torshizi, Samieh Oliaei

**Affiliations:** ^1^ Department of Chemistry, Faculty of Sciences Ferdowsi University of Mashhad Mashhad Iran; ^2^ Department of Plant Production Saffron Institute, University of Torbat Heydarieh Torbat Heydarieh Iran

**Keywords:** adsorption isotherm, anthocyanin adsorption, bentonite, kinetics, saffron tepal

## Abstract

In this study, anthocyanin was extracted from saffron tepals utilizing the ultrasound‐assisted extraction method. The adsorbents of raw bentonite (RB), acid activation of bentonite (AA) thermal activation of bentonite (TA), and acid and thermal activation of bentonite (ATA) were employed to separate anthocyanin from solution. The influence of the operating parameters was evaluated. The isotherm study demonstrated that anthocyanin adsorption on adsorbents could be fitted better by the Langmuir equation than the Freundlich equation. A good agreement between the predicted consequences of the pseudo‐second‐order model and empirical data was provided. Thermodynamic parameters indicated that anthocyanin was adsorbed in an exothermic and physical process. Findings presented that the best adsorption performance of anthocyanin related to ATA with 2.25 mg/g adsorption capacity which was more than 1.55, 1.75, 1.88 mg/g for RB, AA, and TA, respectively. It was due mainly to the increased surface area by both thermal and acid activation of raw bentonite.

## Graphical abstract

The adsorption isotherms and kinetics of the anthocyanin on the modified bentonites extracted from waste of saffron flower was evaluated. The activation of bentonite by both acid and heat exhibited more adsorption than bentonite modified byonly acid or thermal.

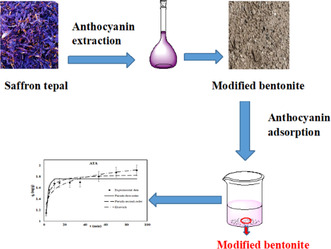

## 1 INTRODUCTION

Food colors are important in assaying the freshness and quality of food to attract the attention of consumer preferences. Due to natural colors being healthier, most government‐regulating agencies have developed the use of natural food colorants instead of synthetic food colorant additives in the food industry since the late 1990s (Francis & Markakis, 1989).

The stigma of the *Crocus sativus* flower, commonly known as saffron, has fascinating properties such as color, flavor, and antioxidant activity. Saffron is most commonly used as an additive in food industry but is less used as a textile dye and an excipient in medical treatments. Only the flower stigma is important in the saffron processing and the other parts are removed. So, 1 kg of saffron is obtained from 68 kg of flowers. Recently, many researchers have been interested in utilizing high‐waste bio‐residues and useless materials. It has been proven that saffron petal extract has “low toxicity” and antiinflammatory, antinociceptive, and antidepressant properties (Moshiri et al., 2006). Other studies have demonstrated that saffron petal extract can be a natural preservative. Water has been found to be the best extraction solvent, with aqueous extracts of saffron petals exhibiting the most antioxidant and antibacterial activities (Abbasvali et al., 2016).

Research has found that phenolic compounds extracted from saffron flower petals have radical scavenging activity (Sánchez‐Vioque et al., 2012). Compounds such as kaempferol glycosides (84.0% of total flavonol content), anthocyanins, and carotenoids were found to be present in the extracts of saffron flower petals (Goupy et al., 2013), and it was also found that Kaempferol in saffron petal extract acts as a tyrosinase inhibitor (Kubo & Kinst‐Hori, 1999). Anthocyanins are responsible for the smart color of the petal (Hemati Kakhki, 2010). By hydrolyzing saffron tepals some anthocyanins and three flavonol aglycones consisting of myricetin, quercetin, andkaempferol were identified by HPLC‐UV.

The anthocyanins extracted from plants may be contaminated with other compounds. Sugars, sugar alcohols, organic acids, amino acids, and proteins are the most common compounds extracted with anthocyanin. Several reactions occur between anthocyanins and the impurities, especially sugar, after extraction. These reactions cause to vary the anthocyanin essence due to formation of bond between anthocyanin and the other molecules and lessen the stability of pigment. Thus, the purification of extracted anthocyanins enhances their range of application and use. The adsorption method has been introduced as an outstanding procedure for eliminating undesirable components. Several papers used sorbents to extract anthocyanins; for example, anthocyanin separation from blueberries employing Amberlite adsorption resins (Buran et al., 2014) or C18, Sephadex, and Amberlite adsorption resins (Castañeda‐Ovando et al., 2009), and extraction of anthocyanins from blueberry by two distinct extraction steps using macroporous resin and Sephadex LH‐20 (Xue et al., 2019). The application of Sephadex LH‐20 for the extraction of anthocyanins from blueberry and cranberry was developed in column chromatography by high‐speed counter‐current chromatography (HSCCC) (Xue, Tan, et al., 2021; Xue, Zhu, et al., 2021). In the new study, the different surface‐modified Fe_3_O_4_ nanoparticles extracted anthocyanins with selectivity and high‐capacity adsorption (Jiang et al., 2024). A mixed‐mode SPE system exploited the benefit of both reversed‐phase and cation exchange interactions (He & Giusti, 2011). This method proved more anthocyanin purification than a solid‐phase extraction (SPE). Researchers considered the anthocyanin tendency to interact with some ions such as Fe^3+^, Cu^2+^, Fe^2+^, Ca^2+^, and Mg^2+^ for complex formation. Thus, materials including these ions were selected to improve extraction (Castañeda‐Ovando et al., 2009; Yoshida et al., 2006). Metal–organic frameworks (MOFs) are structures with large surface areas and pore volumes reported its usage successfully, as adsorbents in the extraction of anthocyanin molecules (Emam & Abdelhameed, 2022).

Bentonite is the class of natural material found in the environment in abundance. In recent years, much attention has been directed on sorbents, especially bentonite owing to its unique properties. Studies have shown that bentonite clay has successfully adsorbed metal ions (Ikram et al., 2002), colors (Gonzalez‐Pradas et al., 1993), and others (Kozar et al., 1992; Olguin et al., 1997). There are a few studies (Das et al., 2018; Deineka et al., 2020) about anthocyanin adsorption by bentonite. Therefore, the purpose of the current work is to study the ability of the modified bentonite to adsorption saffron tepal anthocyanin and optimized conditions for its adsorption. The evaluation of the equilibrium and kinetics of anthocyanin adsorption was done after adsorption. Langmuir, Freundlich, Temkin, and Dubinine–Radushkevich (D–R) equations were exploited to specify the equilibrium isotherm.

## 2 MATERIALS AND METHODS

### 2.1 Materials and instrumentation

The chemicals used in experiments were of analytical grade and were obtained from Sigma‐Aldrich Co. Deionized water (DDW) was used for the preparation of solutions.

An X‐ray fluorometer (XRF) (Philips PW 2404, Netherlands) was used to specify the elemental composition of the bentonite. Absorbance at desirable wavelengths was assessed using a UV–VIS spectrophotometer (Double beam spectrophotometer, Shimadzu, Japan, Model UV‐160A). Fourier transform infrared (FT‐IR) spectra were recorded with a SHIMADZU‐8400S spectrophotometer FTIR. The morphology images of the four types of bentonite were taken by a Philips XL30 Scanning Electron Microscopy (SEM). The porous properties of bentonites were studied by utilizing BET (Brunauer–Emmett–Teller). A BP3001 Benchtop pH meter (Trans instrument, Singapore) was applied to determine pH.

### 2.2 Sample preparation

The saffron flowers were collected from Kashmar in Razavi Khorasan province of Iran. After detaching their stigma, the remaining parts (mainly tepal) were dried in a vacuum oven at 40°C for 36 h. Then, dried tepals were ground into powder using a lab model grinding machine and moved through a 40 mesh sieve. The resulting fine powder was stored in cryotubes at −20°C till further use.

### 2.3 Preparation of adsorbents

#### 2.3.1 Bentonite

The adsorbent used in this study was commercial‐grade bentonite, gray. Initially, it was crushed to fine particle size, ground, and sieved through a 30‐mesh. Then, it was rinsed with deionized water three times to eliminate water‐soluble impurities and other irrelative components from bentonite. The adsorbent was dried at 120°C in an oven for 2 h before further usage.

#### 2.3.2 Acid activation (AA) of bentonite

Natural bentonite was treated by adding 0.5 M HNO_3_. The mixture was refluxed for 2 h at 90°C. The active bentonite was washed several times with deionized water until free NO_3_ was removed and then dried for 2 h in an oven at 80°C for 5 h before use.

#### 2.3.3 Thermal activation (TA) of bentonite

Bentonite was put in a furnace. The temperature was elevated with a heating rate of 10°C/min from ambient to 300°C and kept for 120 min to eliminate the adsorbed water and other impurities. It was cooled to 25°C and stored.

#### 2.3.4 Acid and thermal activation (ATA) of bentonite

Acid and thermal activation (ATA) of bentonite was a combination of two former processes that is AA and TA. Generally, bentonite was activated by 0.5 M HNO_3_ at 90°C in a round‐bottomed flask fitted with a condenser for 2 h. After washing with deionized water, acidic bentonite was heated in a furnace to be calcined at 300°C for 120 min. ATA bentonite was cooled at room temperature and stored.

### 2.4 Methods

#### 2.4.1 Ultrasound‐assisted extraction (UAE) method

Extraction was accomplished by soaking the powdered sample of saffron tepal in an ethanol/water mixture (60: 40 v/v) with a solid to liquid ratio of 1:30 g mL^−1^. The mixture was kept in a glass tube and immersed in a water bath of an ultrasonic extractor coupled to a temperature controller at a temperature of 66°C and a frequency of 23 kHz for 5 min. Then, the extracted solution was filtered with an acetate cellulose membrane filter (0.65 μm). The extract was stored at 4°C for the anthocyanins determination.

#### 2.4.2 Anthocyanin determination

The pH differential method is carried out to evaluate anthocyanin concentration in the extract according to the following equation (Fuleki & Francis, 1968);

(1) $$c=\frac{A\times \mathrm{Mw}\times \mathrm{DF}}{\varepsilon \times L},$$

where *c* is anthocyanins content (mg/L), *A* = [(*A*_530_–*A*_700_)_pH1.0_ – (*A*_530_–*A*_700_)_pH4.5_] in which *A*_530nm_ and *A*_700nm_ are the absorbance readings of anthocyanins at 530 and 700 nm, Mw is the molecular weight of cyanidin‐3‐glucoside (449.2 g/mol), DF is dilution factor, ε is extinction coefficient (26.900 L/cm mol), and *L* is path length of optical glass cell (1 cm).

### 2.5 Adsorption studies

Adsorption experiments on modified bentonite were performed in batch equilibrium mode. The addition of 1 g of adsorbent to the given volume of extracted anthocyanin solution (25 mL) was obtained by successive dilution from a stock solution with deionized water. The McIlvaine buffer solution pH was in the range of 2–7. The resultant was stirred at 45 rpm on a mechanical shaker at 25 ± 1°C. The shaking time was considered in a defined range to complete adsorptive equilibrium. After the equilibration period, the adsorbent was achieved by centrifugation for 30 min to separate the mother liquor from the adsorbent. The clear supernatant was analyzed by UV–visible spectrophotometer to measure the anthocyanin concentration.

Then, adsorbent was washed with deionized water three times, and desorption of adsorbate was performed by 10 mL aqueous solution of ethanol 70% (0.1% v/v HCl) in constant agitation (250 rpm) at 25°C for 60 min. A spectrophotometer assayed anthocyanin concentration in the eluent solution. The ratio of desorption (*D*, %) and recovery (*R*, %) were calculated by the following equations:

(2) $$D=\frac{C_{d}V_{d}}{\left(C_{0}-C_{e}\right)V_{0}}\times 100,$$

(3) $$R=\frac{C_{d}V_{d}}{C_{0}V_{0}}\times 100,$$

where *C*_*d*_ is the concentration of the adsorbate in the eluent solution (mg/mL); *V*_0_ and *V*_*d*_ are the volume of the initial sample and eluent solution (mL), respectively; and *C*_0_ and *C*_*e*_, are the initial and equilibrium concentrations of adsorbate in the solution (mg/mL), respectively. Every test was fulfilled three times and mean values were considered.

### 2.6 Adsorption kinetic modeling

The 1 g adsorbent with 25 mL extract solutions (80 mg/L) and pH 2.6 was mixed in a flask. The flask was stirred at 45 rpm at ambient temperature. An aliquot of supernatant was drawn from the solution every 2 min, up to 200 min. To reveal the mechanism of the adsorption procedure of anthocyanins on the adsorbent, adsorption kinetics was evaluated using pseudo‐first‐order and pseudo‐second‐order kinetic models. The nonlinear equation of the pseudo‐first‐order model is calculated with the following equation:

(4) $$q_{t}=q_{e}\left(1-e^{-K_{1}t}\right)$$

The pseudo‐second‐order model equation is calculated with the following equation:

(5) $$q_{t}=\frac{{q^{2}}_{e}K_{2}t}{1+K_{2}q_{e}t}$$

The nonlinear equation of the Elovich kinetic model is defined with the following equation:

(6) $$q_{t}=\frac{1}{\beta }\ln\left(\alpha \beta \right)+\frac{1}{\beta }\ln\left(t\right)$$

where *k*_1_ is the rate constant of the pseudo‐first model, *k*_2_ is the rate constant of pseudo‐second‐order model, *q*_*e*_ (mg/g) is the adsorption capacity of adsorbent at equilibrium, and *q*_*t*_ (mg/g) is the amount of anthocyanin adsorbed at time *t*. *α* (mg/g min) is the initial sorption rate, and *β* (g/mg) is correlated to the extent of surface coverage and activation energy for chemisorption.

### 2.7 Isotherm studies

The experiments were carried out into an Erlenmeyer flask with 1 g adsorbent and 25 mL extract solutions at diverse concentrations. The flask  was shaken at 45 rpm for 150 min in a vibrator shaker at room temperature. The spectrophotometric method assessed initial and equilibrium concentrations at mentioned temperatures. From Langmuir (Equation 7), Freundlich (Equation 8), Temkin (Equation 9) and (D–R) (Equation 10) models were exploited to identify adsorption isotherm of anthocyanin on the adsorbent according to the Equations ((7), (8), (9), (10)):

(7) $$q_{e}=\frac{q_{m}K_{L}C_{e}}{1+K_{L}C_{e}}$$

(8) $$q_{e}=K_{F}{C_{e}}^{\frac{1}{n}}$$

(9) $$q_{e}=B\;ln(AC_{e})=\left(\frac{\mathrm{RT}}{b_{T}}\right)\;\ln(AC_{e})$$

(10) $$q_{e}=q_{m}\exp\left(-{\beta \varepsilon }^{2}\right),$$

where *q*_*e*_ is adsorption capacity at equilibrium (mg/g), *C*_*e*_ is equilibrium concentration of anthocyanin in solution at equilibrium (mg/L), *q*_*m*_ is extreme adsorptive capacity (mg/g), *K*_*L*_ is Langmuir adsorption equilibrium constant (L mg^−1^), *K*_*F*_ is Freundlich adsorption equilibrium constant (mg/g)(L/mg)^1/n^, 1/*n* is an experimental constant indicative the importance of adsorption driving force, *B* or RT/*b*_*T*_ (J/mol) is Temkin constant related to heat sorption, *A* (L/mg) is equilibrium binding constant, *R* (8.314 J/mol K) is universal gas constant, and *T* (K) is absolute solution temperature, *β* is a constant related to the sorption energy, *ε* is Polanyi potential.

### 2.8 Thermodynamic studies of adsorption

Thermodynamic factors give adsorption characteristics of adsorbent considering two changes in structure and inherent energy of adsorbent after adsorption. Also, these parameters help to forecast the adsorption mechanism. Gibbs free energy change (KJ/mol) is estimated by Equation (11)

(11) $$∆G^{0}=-\mathrm{RT}\ln\left(K_{D}\right)$$

where *R* is the universal gas constant (8.314 J/mol K), *T* is the absolute temperature (*K*), and *K*_*D*_ is the thermodynamic equilibrium constant, which is the fraction of adsorbed anthocyanin on the adsorbent to the amount remaining anthocyanin in solution. The Δ*H* ^0^ and Δ*S* ^0^ parameters can be estimated by the Clausius–Clapeyron equation, according to Equation (12).

(12) $$\ln\left(K_{D}\right)=-\frac{∆H^{0}}{\mathrm{RT}}+\frac{∆S^{0}}{R}$$

### 2.9 Diffusion model

Transportation of the solute onto the adsorbents is ascertained with the adsorption mechanisms of the solute. The mechanism is the basis of the intra‐particle diffusion model proposed to have a better appreciation of the adsorption process. According to this model, adsorption ability is directly related to the half‐power of time. The most commonly useful intra‐particle diffusion equation in the adsorption of biomaterial is obtained by Weber and Morris as follows (Weber & Morris, 1962):

(13) $$q_{t}=K_{t}t^{0.5}+C$$

where *q*_*t*_ is the adsorption capacity of adsorbent (mg/g), *k*_*t*_ is the intra‐particle diffusion rate constant (g/mg min^0.5^), and *C* is the intercept.

## 3 RESULTS AND DISCUSSION

### 3.1 Characterization of adsorbents

The chemical analysis of the bentonite used in the present research was as follows: 61.7% SiO_2_, 3.4% MgO, 14.5% Al_2_O_3_, 0.9% K_2_O, 0.3% CaO, 2.3% Fe_2_O_3_, 0.5% Na_2_O, 0.6% TiO_2_, and 15.3% the other compounds. Bentonite cation exchange capacity (CEC) was determined using the methylene blue method (Taylor, 1985) 85 meq/100 g. The average BET surface area of RB, TA, AA, and ATA was at 23, 42, 67, and 81 m^2^ g^−1^, respectively.

**FIGURE 1:**
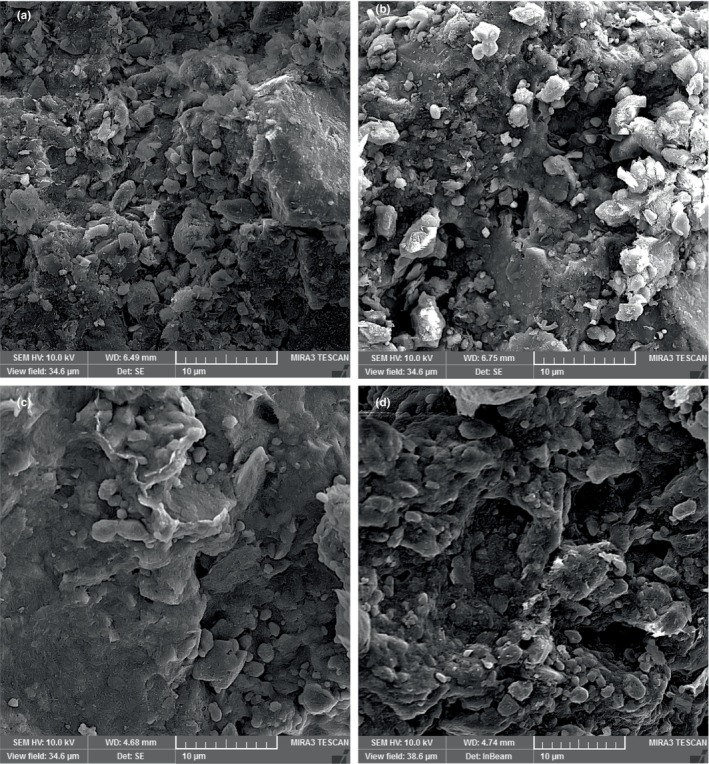
Scanning electron microscopy (SEM) images of applied bentonites as adsorb (a) Raw bentonite (RB), (b) Thermal activation (TA), (c) Acid activation (AA), and (d) Acid and termal activation (ATA).

The morphology of adsorbents is shown in Figure 1. The structural images of adsorbents illustrated that the structure of RB has lower porous than the rest of the structures. The heating of bentonite disturbed the ordered structure of RB and made new pores in the structure of TA. Furthermore, removing impurities or replacing cations with H^+^ ions enhanced the pores in the acidic treatment of RB. The pros of the most porosities were seen in ATA due to acidic and thermal activations.

### 3.2 Effect of pH on adsorption

Initial pH affects the molecular form of anthocyanin in the aqueous environments. The structural transformations that is flavylium cation (AH^+^), colorless hemiketal carbinol pseudobase (B), purple neutral quinoidal base (N), and anionic quinoidal base (A) depend on pH solution. In an acidic environment, anthocyanin is in red flavylium cation form, while the blue quinoidal base form is observed in a basic environment.

The pH effect was investigated using McIlvaine buffer solutions over an initial pH range of 2–7. The adsorption efficiency remained slight as the pH changed from 2 to 7. It may be explained through electrostatic interaction and chemical bonds amid bentonite and anthocyanin. There are durable and pH self‐reliance negative charges in bentonite derived from isomorphic substitutions of ions with less charge like Mg^2+^, Fe^2+^, or Mn^2+^ in place of Al^3+^ in the octahedral layer and Al^3+^ or sometimes Fe^3+^ substitutes in place of Si^4+^ in the tetrahedral layer (Javed et al., 2018). The positively charged flavylium structural form (Brouillard, 1983) and bentonite in the negatively charged surface structure would likely interact electrostatically at low pH. The most important constituent in bentonite is SiO_2_ with a zero point charge value (pH zpc) equivalent to 2.2 (Mall et al., 2005), indicating that this constituent can interact with positively charged adsorbates at pH more than pH zpc. The excess H^+^ ions at low pH are contrary to flavylium cations because a competitive environment between the H^+^ ions and flaviyium cations occurs on adsorption sites with negative charges. So the adsorption mechanism may be hardly affiliated with ionic interaction. Predominantly, anthocyanin is in flavylium form at low pH values and it decreases as the pH value rises, hydration and proton transfer reactions can occur with the creation of lots of different chemical structures. Hence, the pH value for subsequent studies was the when equilibrium between flavylium cation and carbinol pseudobase is attained at a pH of 2.6.

### 3.3 Optimization of the eluent

Initially, the eluent solution was considered an acidic aqueous (0.1% HCL) solution in the desorption step, indicating anthocyanin desorbed slightly into the eluent solution. Then various ratios of ethanol solutions (10%, 30%, 50%, 70%, 90%, and 100% v/v) containing 0.1% HCL were selected as eluent. The desorption of anthocyanin was utmost with the ratio of desorption (D) more than 95% in a 70% ethanol and 0.1% HCL solution. As an aqueous solution with acidic pH cannot desorb anthocyanin of adsorbents solely, ethanol solution improved the desorption process somewhat very much, resulting in the electrostatic interaction existing between anthocyanin and eluent solution as well as the polarity of eluent solution may contribute in anthocyanin desorption process.

### 3.4 Effect of contact time

**FIGURE 2:**
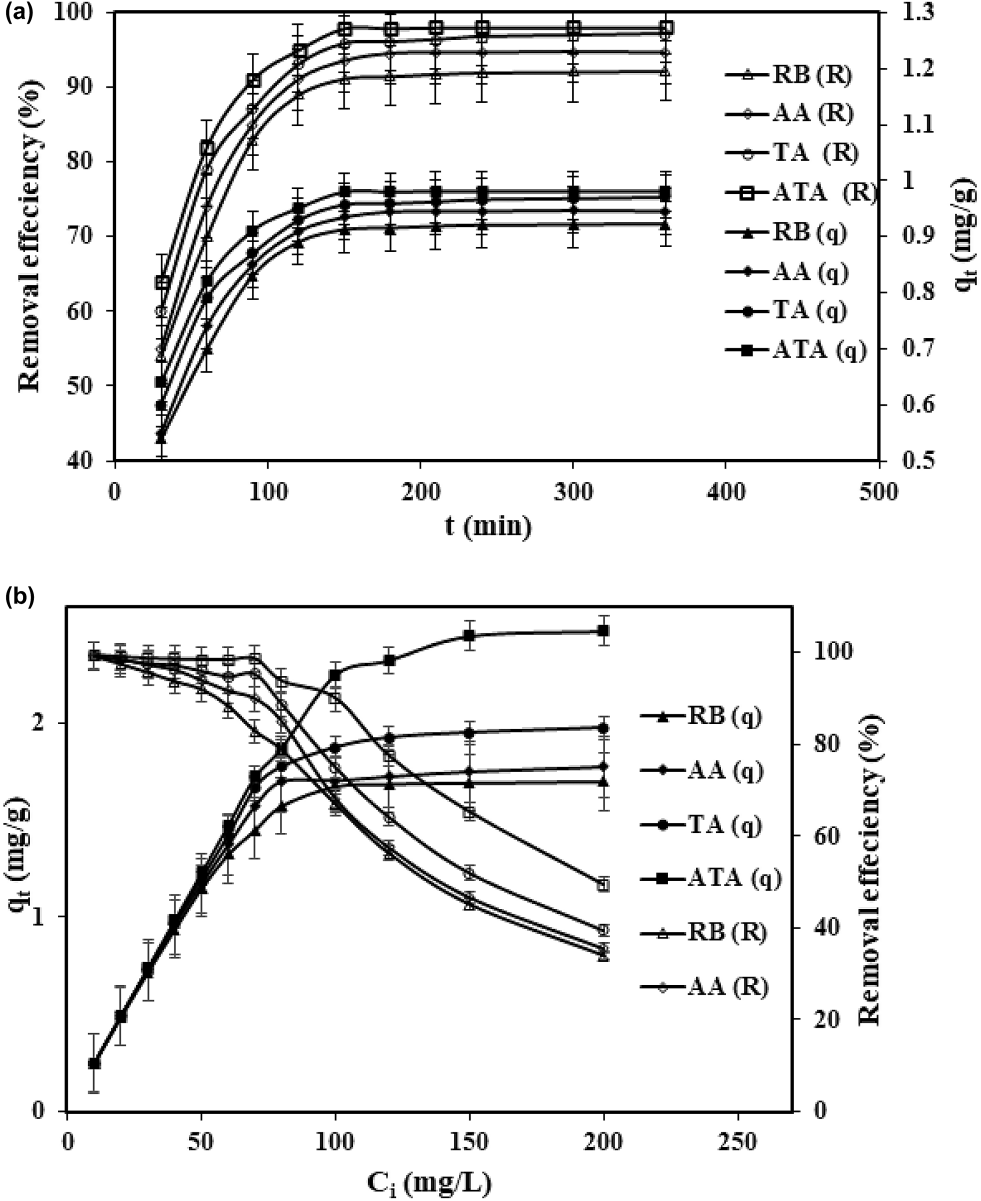
Optimization of parameters (a) the effect of contact time on the adsorption of anthocyanin (1 g adsorbent was added to a anthocyanin solution 40 mg/L with pH 2.6 at temperature 298 K and shaken at different times) and (b) the effect of initial anthocyanin concentration of the adsorption on the adsorbent (1 g adsorbent was added to a anthocyanin solution with different concentrations in pH 2.6 and shaken within 180 min at temperature 298 K).

The adsorption of anthocyanin depends on the contact time. A set of sample flasks containing 25 mL anthocyanin solution (40 mg/L) and bentonite (1 g) was prepared and shaken at different times to investigate the effect of contact time. As shown in Figure 2a, the adsorbents that have more contact time with anthocyanin molecules, the more anthocyanin molecules removed. The adsorption was up to 90% before 1 h for all adsorbents. The rather fast adsorption of anthocyanin onto the adsorbent emphasized the high affinity between the adsorbant and bentonite. In interval time 2–3 h adsorbents were removed from 90% to 98% anthocyanin (the values of adsorption capacity and percent of recovery were (0.92 mg/g; 90%), (0.94 mg/g; 93%); (0.98 mg/g, 97%), and (0.99 mg/g; 98%) for RB, AT, AA, and ATA, respectively) indicating the adsorption rate was much less than the initial times and the adsorption reaction reached equilibrium at a contact time of 3 h. The reason was that bentonite had many available active sites in the first adsorption process. Over time, anthocyanin molecules occupied sites available until they reached an equilibrium, meaning the amount adsorbant did not change considerably and the anthocyanin molecules saturated the bentonite surface. Among the adsorbents, the highest adsorption rate for the anthocyanin concentration of 40 mg/L was possibly for ATA due to being of more adsorption sites that can adsorb anthocyanin molecules from solution.

### 3.5 Effect of initial anthocyanin concentration

Anthocyanin adsorption on the bentonites shown in Figure 2b indicated the effect of initial anthocyanin (*C*_*i*_) on bentonites under adsorption circumstances: volume of solution (25 mL), T (25°C), pH (2.6), contact time (3 h) and bentonite dosage (1 g). There was a direct relationship between the anthocyanin adsorption and the initial anthocyanin concentration. The more initial anthocyanin concentration the more anthocyanin adsorption indicating predomination of mass transfer resistance by mass gradient pressure force moved anthocyanin through bulk solution towards the bentonite surface.

The adsorption rate of initial anthocyanin concentration was high at first and gradually decreased to meet close to zero at 100 mg/L. Having empty sites on the adsorbent, raised interaction between adsorbent and adsorbate at the early times. After a lapse of time, occupation of residuary empty surface sites was tough as repulsive forces from similar charges of anthocyanin on the surface of bentonite and in the solution phase.

In Figure 2b, raw bentonite showed the minimum values *q*_*e*_ (1.55 mg/g), R (66%), while maximum values of the q_e_ were for the ATA (2.25 mg/g), R (98%) followed by AT (1.88 mg/g), R (74%) and AA (1.75 mg/g), R (67%). The thermal treatment of bentonite probably led to the oxidation of organic matter sticking to the external surface of bentonite. By heating, available active sites increased dramatically which could affect the improvement of the adsorption procedure. On the contrary, in mild acid treatment leaching of some divalent and trivalent ions in octahedral layers resulted in several amorphous silica phases with an increase in the Si–OH bonds (Panda et al., 2010), however, the surface area of bentonite was raised (Kooli et al., 2015). It may be reasonable that enhanced surface area outweighs the development in negative charges on the surface of bentonite in mild acid treatment so a higher adsorption capacity was observed for AA than RB.

### 3.6 Kinetic models

**FIGURE 3:**
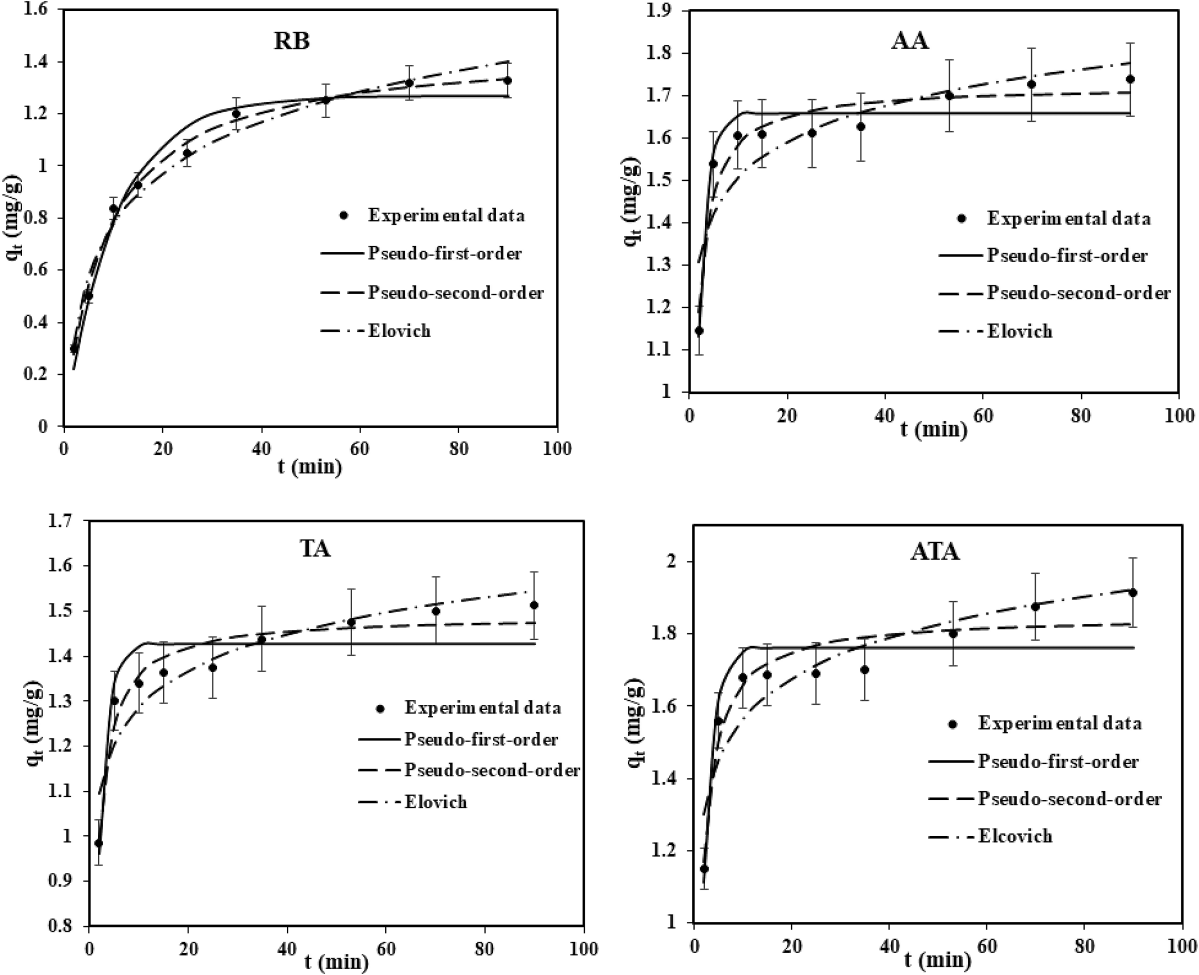
Kinetic moles of Pseudo‐first‐order, pseudo‐second‐order, and Elovich for anthocyanin adsorption onto adsorbents (1 g adsorbent was added to a anthocyanin solution 80 mg/L with pH 2.6 and shaken within different times at temperature 298 K).

The rate of adsorption can influence the evaluation and quality of an adsorbent. The adsorption rate constant (*K*_1_, *K*_2_), the maximum adsorption capacities *q*_e_, and correlation coefficients for adsorbents calculated from the pseudo‐first and pseudo‐second order model were displayed in Table 1. These data were acquired from both models, pseudo‐first, and pseudo‐second order shown in Figure 3.

**TABLE 1:** Pseudo‐first order, pseudo‐second order, intra‐particle diffusion, and Elovich and Boyd model parameters of adsorbents for the adsorption anthocyanin.

	Pseudo‐first order			Pseudo‐second order		
	*K*_1_ (1/min)	*q*_*e*_ (mg/g)	*R* ^2^	*K*_2_ (g/mg min)	*q*_*e*_ (mg/g)	*R* ^2^
RB	0.0309	0.8382	0.970	0.08156	1.4573	0.992
AA	0.02833	0.3802	0.830	0.3335	1.5375	0.940
TA	0.04122	0.4657	0.903	0.2896	1.7665	0.930
ATA	4.2 × 10^−5^	1.7133	0.826	0.1521	1.9531	0.915

	Intra‐particle diffusion					
	*K*_id,1_ (mg/g min^1/2^)	*C*_1_	*R* ^2^	*K*_id,2_ (mg/g min^1/2^)	*C_2_*	*R* ^2^
RB	0.1629	0.1939	0.912	0.0266	1.0725	0.864
AA	0.0702	1.0141	0.903	0.0216	1.3175	0.949
TA	0.0777	1.1795	0.924	0.0145	1.5985	0.951
ATA	0.0966	1.1299	0.967	0.0384	1.5371	0.920

	Boyd model		Elovich		
	*Bt*	*R* ^2^	*ß* (g/mg )	*α* (mg/g min)	*R* ^2^
RB	0.0308	0.938	3.5063	0.4309	0.976
AA	0.0299	0.993	8.071	336.0600	0.851
TA	0.0280	0.946	7.1994	501.0890	0.750
ATA	0.0411	0.990	5.3619	53.1841	0.836

The calculated data from pseudo‐first‐order kinetic models for whole adsorbents did not match well with the empirical data. Whereas, pseudo‐second‐order models fit well implying the pseudo‐second‐order models had better correlation coefficient values than pseudo‐first‐order kinetic models. The maximum adsorption capacities *q*_*e*_ measured from the pseudo‐second model were in excellent compliance with the empirical values. In this model, the values of the equilibrium adsorption capacity were compared with the data of the experimental adsorption capacity for an initial anthocyanin concentration of 80 mg/L. Thus, it was alleged that the pseudo‐second order model properly described the adsorption process of anthocyanin molecules on the bentonites. This model infers the postulation that chemisorption has a limited adsorption rate that occurs basically through sharing electrons between adsorbent and adsorbant (Duarte Neto et al., 2018; Ho & McKay, 1998). Similar outcomes for the adsorption of anthocyanin have also been reported by other papers (Norman et al., 2016; Toor & Jin, 2012).

Elovich model is useful for elucidating the chemosorption on heterogeneous surfaces (Table 1). The Elovich kinetic model (Figure 3) did not represent the relatively good compliance between the kinetic data and the Elovich model. Afterward, the interpretation of the Elovich model was not appropriate for predicting the anthocyanin adsorption on adsorbents.

### 3.7 Adsorption mechanism

The pseudo‐first‐order and pseudo‐second‐order kinetic models are unable to justify the diffusion mechanism. Weber and Morris (Weber & Morris, 1962) considered a reasonable diffusion mechanism of components onto the prepared adsorbents. The adsorbate diffusion in a bulk solution into sorbent takes place in four consecutive steps: (i) diffusion of adsorbate in bulk solution to bordering film encircling the adsorbent particles (film diffusion); (ii) diffusion of adsorbate to outer particles (external diffusion); (iii) diffusion of adsorbate within internal sites (surface diffusion or pore diffusion); and (iv) the reaction of adsorption and desorption of adsorbate including physicochemical interaction, ion exchange, precipitation or complexation (Gerçel et al., 2007). Thus, the diffusion process is controlled by one or some of the above steps.

**FIGURE 4:**
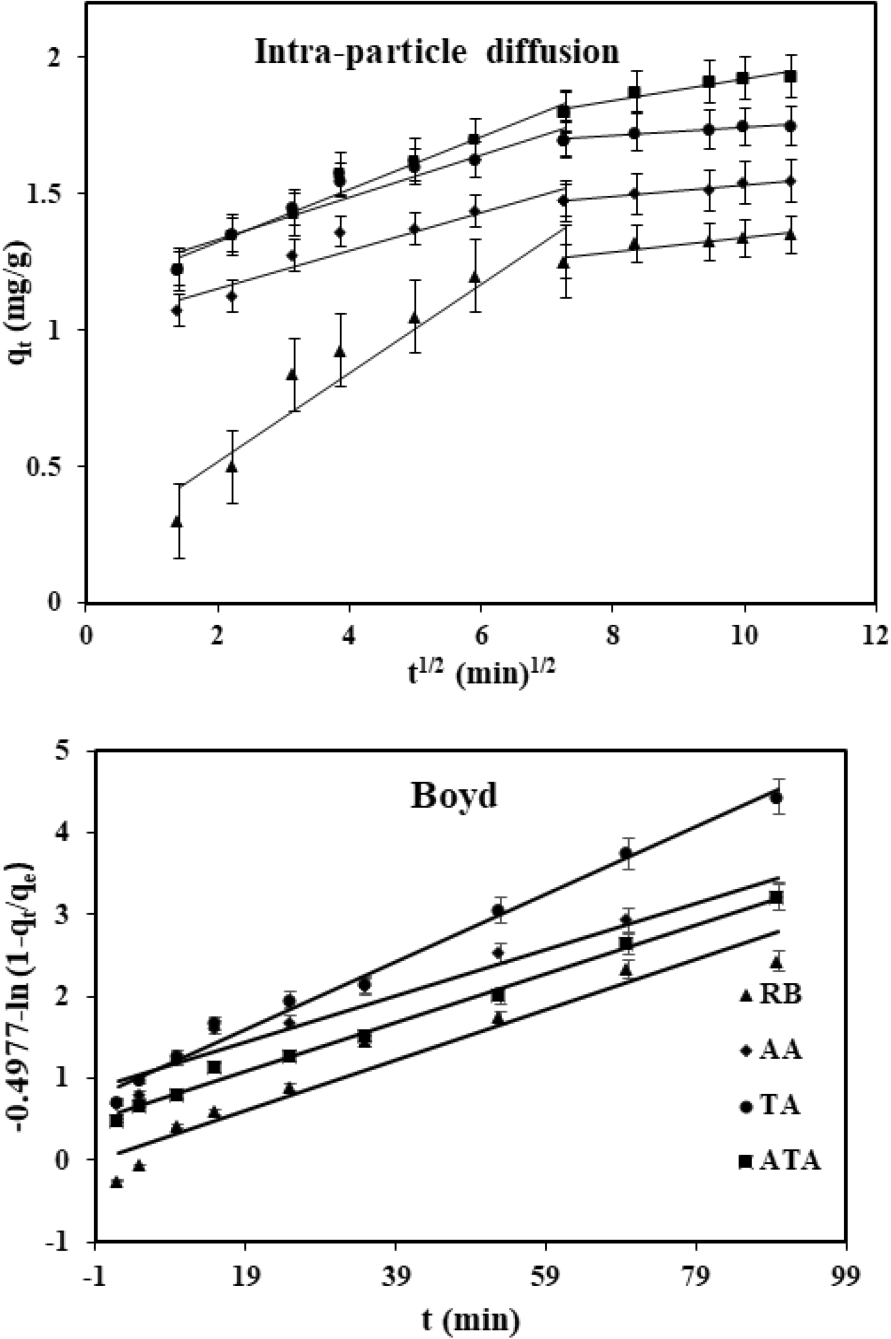
Intra‐particle diffusion, and Boyed models for anthocyanin adsorption onto adsorbents (1 g adsorbent was added to a anthocyanin solution 80 mg/L with pH 2.6 and shaken within different times at temperature 298 K).

Figure 4 illustrated that plots of *q*_*t*_ against *t* ^0.5^ were contained from three distinctive linear regions. The first stage of the linear region with a high slope was associated with external surface adsorption or the instantaneous adsorption accomplished during 90 min the following one with moderate clarified intra‐particle diffusion in the second linear region. As seen in Figure 4 intra‐particle diffusion contributed to the rate mechanism. However, the other mechanisms restricted the adsorption rate, because extrapolating linear straight lines cannot reach the origin and other mechanisms were together with intra‐particle diffusion. The increase of the constant *C* values as an order of ATA > TA > AA > RB under this studied situation can be ascribed to an extension of the thickness of the bordering film resulting in higher adsorption capacities (Özcan et al., 2005).

To further analyze the actual kinetic data affecting the rate‐controlling step of the adsorption, the Boyd model (Boyd et al., 1947) asserted:

(14) $$\mathrm{Bt}=-0.4977-\ln\left(1-\frac{q_{t}}{q_{e}}\right)$$

where *Bt* is the mathematical function that includes the ratio of anthocyanin molecules adsorbed on bentonites (*q*_*t*_/*q*_*e*_), *q*_*t*_ is quantities of anthocyanin molecules adsorbed on bentonite at time *t*, and *q*_*e*_ is quantities of anthocyanin molecules adsorbed on bentonite at equilibrium.

Figure 4 displayed linear profiles of plots −0.4977 − ln(1 − *q*_*t*_/*q*_*e*_) versus *t* did not pass through the origin indicating the pore diffusion was not the rate‐limiting step and external transport mainly governed controlled mechanism of anthocyanin molecules adsorption on adsorbents.

**FIGURE 5:**
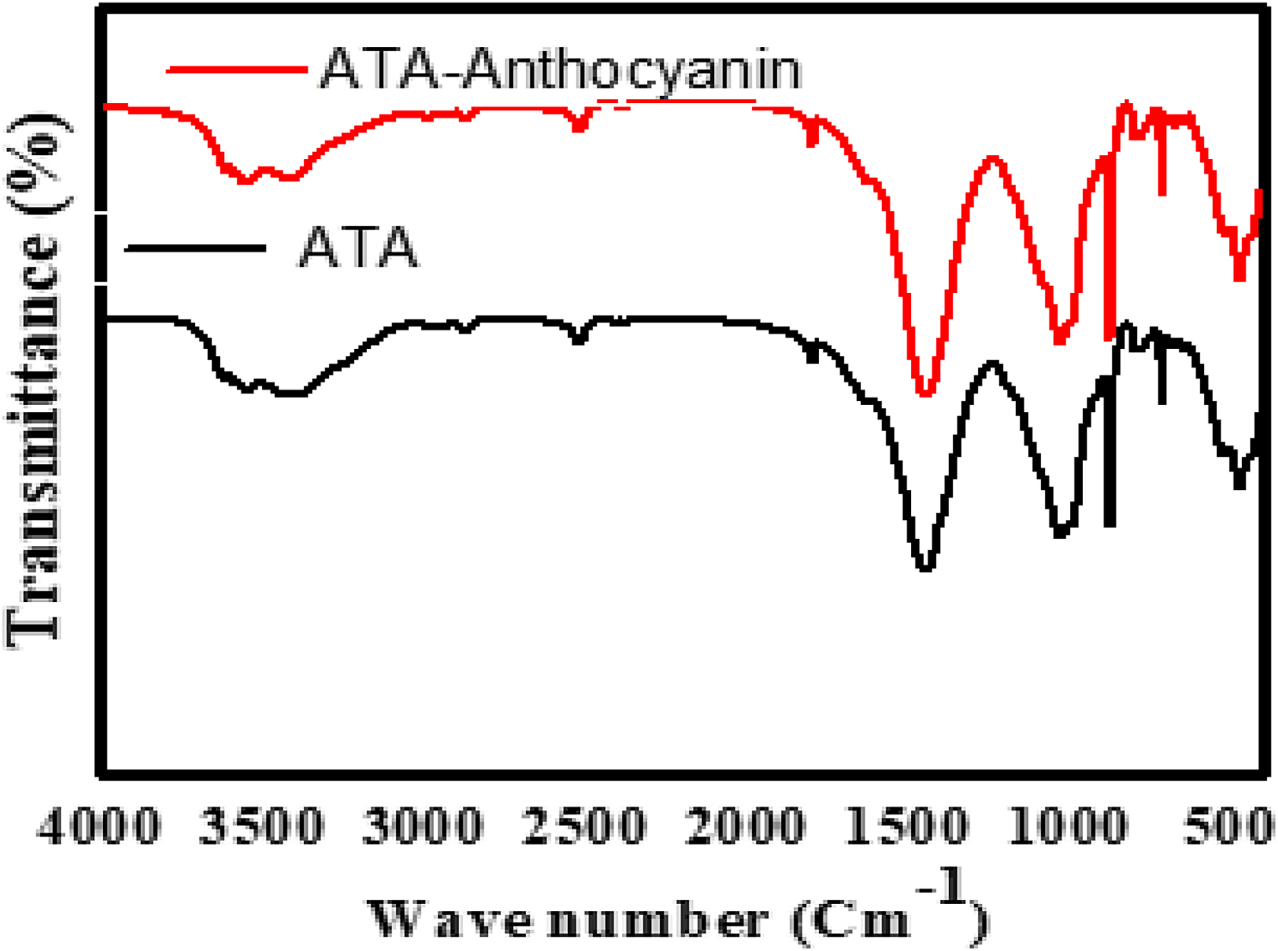
Fourier transform infrared (FT‐IR) spectra of acid and thermal activation (ATA) of bentonite and acid and thermal activation of bentonite with adsorbed anthocyanin (ATA‐anthocyanin).

FTIR spectra of ATA and ATA along with anthocyanin are shown in Figure 5. The bands at 3400 and 1623 cm^−1^ were H‐O‐H stretching and bending vibrations of adsorbed water, respectively. The located peaks at 1454, 1801, and 2987 cm^−1^ are attributed to the CO_2_ environment. The band seen at 991 cm^−1^ indicated the Si‐O group in‐plane vibration. The Al‐Al‐OH, Al‐Fe‐OH, and Al‐Mg‐OH were situated in 912, 873, and 798 cm^−1^. After the adsorption of anthocyanin on ATA has not created any new band, there is no strong chemical interaction between ATA and anthocyanin. However, trivial shifts to high wavenumbers in region 3550–3620 cm^−1^ are observed that may arise from the stretching and bending vibrations of O‐H groups in anthocyanin adsorbed on ATA.

### 3.8 Adsorption isotherms

Adsorption isotherms are essential to understanding how the distribution of adsorbates is balanced in a system containing two liquid–solid phases. The adsorption isotherm is determined based on the best correlation coefficient of the equilibrium curves, and by using it, many parameters influencing adsorption such as the adsorption mechanism, and the tendency of the adsorbent to adsorbant are identified. Four well‐known models, the Langmuir, Freundlich, Temkin, and Dubinin–Radushkevich (D–R) isotherms, were studied to clarify interactions between the adsorbent and adsorbant.

The Langmuir isotherm model is well‐known and assumes that adsorption is a process that happens via a single‐adsorbant mechanism on the homogeneous outer coverage of adsorbent with identical adsorption activation energies. The adsorbate molecules create a monolayer on the surface of the adsorbent without having any interaction among themselves. Thus, after lapsing a time the surface of adsorption attains a saturation value and no more molecules are located on it. Conversely, the Freundlich isotherm is an empirical equation to describe the multi‐layer adsorption process on a heterogeneous coverage.

**FIGURE 6:**
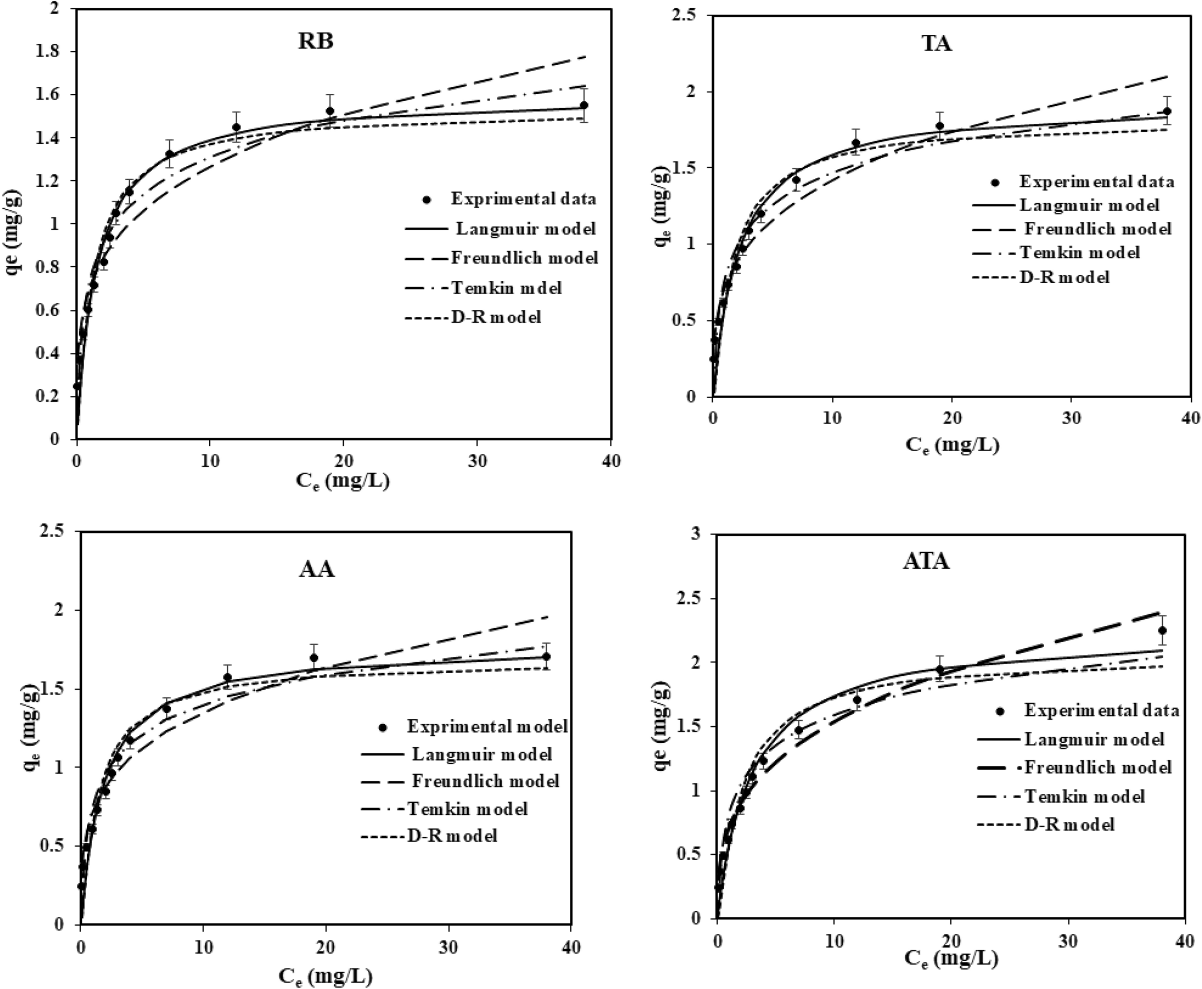
**P**lots of adsorption isotherms Langmuir and Freundlich, Temkin, and D–R for anthocyanin adsorption onto the sorbents (1 g adsorbent was added to a anthocyanin solution with different concentrations in pH 2.6 and shaken within 150 min at temperature 298 K).

Adsorption information was evaluated more by Langmuir and Freundlich isotherm equations. Table 2 shows obtained results of the Freundlich and Langmuir model parameters for bentonites. From Figure 6, it could be deduced that experimental data conformed well with the Langmuir model than the Freundlich model. In the Langmuir model, computed extreme monolayer adsorption (*q*_*m*_) was close to equilibrium adsorption from experiments (*q*_*e*_). From Table 2, quantities *n* which are more than 1, imply effective adsorption intensity.

**TABLE 2:** Different isotherm parameters for anthocyanin adsorption onto adsorbents.

	Isotherm parameters							
	Langmuir		Freundlich		Temkin		D–R	
RB	*K*_*L*_ (L mg^−1^)	0.7239	*K*_*f*_ (mg/g)(L/mg)^1/n^	0.6386	*A*(L mg^−1^)	0.02045	*q*_*m*_ (mmol g^−1^)	1.3 × 10^−5^
	*q*_max_ (mg g ^−1^)	1.6595	*n*	3.1381	*b*_*T*_ (KJ mol^−1^)	10.5510	*ᵦ* (mol^2^ KJ^−2^)	2.1000
	*R* ^2^	0.961	*R* ^2^	0.918	*B*	0.2464	*E*_*a*_ (KJ mol^−1^)	15.4303
					*R* ^2^	0.949	*R* ^2^	0.935
AA	*K*_*L*_ (L mg^−1^)	0.9606	*K*_*f*_ (mg/g)(L/mg)^1/n^	0.7353	*A* (L mg^−1^)	0.02088	*q*_*m*_(mmol g^−1^)	1.7 × 10^−5^
	*q*_max_ (mg/g)	1.7665	*n*	2.9206	*b*_*T*_ (KJ mol^−1^)	8.7085	*ᵦ* (mol^2^ KJ^−2^)	2.2000
	*R* ^2^	0.962	*R* ^2^	0.933	*B*	0.2845	*E*_*a*_ (KJ mol^−1^)	15.0756
					*R* ^2^	0.944	*R* ^2^	0.924
TA	*K*_*L*_ (L mg^−1^)	1.0054	*K*_*f*_ (mg/g)(L/mg)^1/n^	0.8268	*A*_*T*_(L mg^−1^)	0.0194	*q*_*m*_ (mmol g^−1^)	2.4 × 10^−5^
	*q*_max_ (mg/g)	1.9865	*n*	2.6582	*b*_*T*_ (KJ mol^−1^)	7.5330	*ᵦ* (mol^2^ KJ^−2^)	2.4000
	*R* ^2^	0.964	*R* ^2^	0.950	*B*	0.3289	*E*_*a*_ (KJ mol^−1^)	14.4338
					*R* ^2^	0.940	*R* ^2^	0.919
ATA	*K*_*L*_ (L mg^−1^)	1.3258	*K*_*f*_ (mg/g)(L/mg)^1/n^	1.1735	*A* (L mg^−1^)	0.0176	*q*_*m*_ (mmol g^−1^)	6.2 × 10^−5^
	*q*_max_ (mg/g)	2.4307	*n*	2.0513	*b*_*T*_ (KJ mol^−1^)	5.3477	*ᵦ* (mol^2^ KJ^−2^)	3.0000
	*R* ^2^	0.957	*R* ^2^	0.982	*B*	0.4633	*E*_*a*_ (KJ mol^−1^)	12.9099
					*R* ^2^	0.919	*R* ^2^	0 .900

According to Langmuir and Freundlich equations, *k*_*f*_ and *k*_*L*_ values increase in the order of ATA > TA > AA > RB. It shows relative adsorption capacity was amplified by modifying raw bentonite with thermal and acid activation.

The negative surface charge of bentonite results from substitutions in either the octahedral sheet (displacement of low charge cations such as Mg^2+^, Fe^2+^, or Mn^2+^ for Al^3+^) or tetrahedral sheet (replacement of Al^3+^ or sometimes Fe^3+^ for Si^4+^) culminating in net negative charges on the bentonite. These negative charges are compensated by the sorption of exchangeable cations such as Na^+^, K^+^, Ca^2+^, and so forth (Sposito, 1984). The main adsorption mechanism is due to exchange reactions occurring between the positive charge of anthocyanin molecules and cations Na^+^, K^+^, Ca^2+^, and so on into interlayer space. It has been suggested that harsh acidic treatment can eliminate many Al^3+^ ions from the octahedral sheets and increase amorphous silica phases (Panda et al., 2010). In contrast, mild acidic treatment can create porosity and disaggregate and delaminate bentonite layers (Rodriguez et al., 1995) so that it can contribute to the increment of the surface area because some cations in large sizes have been replaced by H^+^ in small sizes (Teng & Lin, 2006). Thus, the structural virtues of the bentonite particles have been improved which favors the adsorption of anthocyanin. Thermal activation of bentonite removes non‐clay matter and makes active sites. Moreover, the calcination delaminates the aluminosilicate sheets, promoting a cross‐linking structure with increasing mesoporosity of bentonite and developing the surface area (Mahboub et al., 2006). It was conceived that more anthocyanin was held in active space between the silicate layers when bentonite was active through thermal or acid. Generally, thermal and acid treatment of bentonite raised chemical stability and was prone to adsorb anthocyanin molecules with having more active sites. For this reason, ATA had the most adsorption capacity for anthocyanin molecules in the solution. The adsorption process likely proceeds via the physisorption mechanism.

To get favorability and feasibility of the adsorption system, the dimensionless constant separation factor or equilibrium parameter, *R*_*L*_ was considered. By Langmuir isotherm. Equation (7) can take the separation factor *R*_*L*_:

(15) $$R_{L}=\frac{1}{1+K_{L}C_{i}}$$

where *K*_*L*_ is the Langmuir constant and Ci is the initial concentration of anthocyanin molecules. The values of *R*_*L*_ determine the shape of the isotherm. The isotherm is either unfavorable (*R*_*L*_ > 1), linear (*R*_*L*_ = 1), favorable (0 < *R*_*L*_ < 1) or reversible (*R*_*L*_ = 0). According to Equation (15) and analysis from the experimental data, all the quantities of *R*_*L*_ towards adsorption of anthocyanin molecules on adsorbents were less than 0.08 showing the adsorption process was favorable. The lowest *R*_*L*_ of sorbents related to ATA shows that the anthocyanin molecule adsorption on this adsorbent was more favorable than the others.

The Temkin isotherm constants *A*, *B*, and *b*_*T*_ were computed for adsorbents. *b*_*T*_ corresponds to the heat of adsorption of anthocyanin on the adsorbents. The values *b*_*T*_ were in the range of 10.5510 to 5.3477 KJ/mol. As seen in Table 2 by increasing *q*_max_, the value of *b*_*T*_ decreased and as a result, the interaction between the adsorbent and the adsorbent becomes weaker.

Experimental data matched well with the D‐R isotherm model (Table 2). The description of the adsorption process into a solution meaning the transition molecules from solution to adsorbent surface could be explained by sorption mean free energy (*E_a_=(1/2β)^1/2^*). The value of *E*_*a*_ in the range of 1–8 kJ/mol specifies physical adsorption, a value *E*_*a*_ more than 8 up to 16 kJ/mol pronounces an ion exchange process and a value *E*_*a*_ lies between 16 and 40 kJ/mol elucidates chemical adsorption as dominant mechanism. The mean sorption energies were estimated to be 15.4303, 15.0756, 14.4338, and 12.9099 kJ/mol for RB, AA, TA, and ATA, respectively. It showed the main adsorption mechanism in the adsorption of anthocyanin onto adsorbents was ion exchange, accompanied by physical interaction. It evidenced that thermal and acid activation of bentonite significantly decreased the *E*_*a*_, suggesting that more physical interaction between bentonite and anthocyanin grew.

### 3.9 Thermodynamic studies

The influence of temperature on anthocyanin molecule adsorption was tested under the optimal conditions at 293, 303, 313, and 323 K presented in Table 3. The values of Δ*G* ^0^, Δ*H* ^0^, and Δ*S* ^0^ for adsorbents are given in Table 3. The calculation demonstrated that values of Δ*H* ^0^ were negative, indicating the adsorption process was exothermic, as verified in Table 3. The adsorption of anthocyanin molecules on the adsorbents dropped slightly as the temperature rose from 293 to 323 K. Adsorption of anthocyanin on adsorbents has in physical nature based on physisorption has adsorption enthalpy change in the range from −20 to 40 kJ/mol, while the high values of adsorption enthalpy change consisting from −400 to −80 kJ/mol associated with chemisorption (Lian et al., 2009). Among the adsorbents, the ATA had the most enthalpy change, implying that it had the most adsorption strength between adsorption and adsorbant. According to similar results reported for anthocyanin adsorption on resins (Chen et al., 2016) and amberlite XAD7 (Das et al., 2018), raising the temperature enables to break the weak interactions such as hydrogen bonds and Vander Waals formed between adsorbent and adsorbate. Negative quantities of free energy change (Δ*G* ^0^) described the feasibility and the spontaneous character of the adsorption procedure with the great tendency of anthocyanin adsorption on bentonite (Table 3). Besides, the negative quantities Δ*S* ^0^ propose an irregular reduction in randomness at their adsorbent‐solution boundary throughout the adsorption process, and the internal structure of the adsorbents remained without any noticeable variations. (Alkan et al., 2007).

**TABLE 3:** Parameters of thermodynamic model for the adsorption of anthocyanin onto adsorbents.

	Temperature (K)	∆*G* ^0^ (kJ mol^‐1^)	∆*H* ^0^ (kJ mol^−1^)	∆*S* ^0^ (J mol^−1^ K^−1^)
RB	293	−2.6762	−11.2164	−29.1289
	303	−2.3792		
	313	−2.1432		
	323	−1.7812		
AA	293	−3.2275	−11.3918	−27.7343
	303	−3.0439		
	313	−2.7219		
	323	−2.4045		
TA	293	−3.5322	−13.0305	−32.4155
	303	−3.1884		
	313	−2.9287		
	323	−2.5363		
ATA	293	−3.6938	−13.1785	−32.3972
	303	−3.3681		
	313	−2.9996		
	323	−2.7395		

### 3.10 Comparison of performance of current study with other methods

The adsorption performance of anthocyanin on some different adsorbents in terms of adsorption capacity was evaluated (Table 4). An adsorbent such as bentonite is cost‐effective compared to other adsorbents such as resin for anthocyanin adsorption. However, most resins would show a higher adsorption capacity towards anthocyanin adsorption particularly if modification is implemented on the resin surface. Recently, the modified bentonite has been applied for anthocyanin adsorption, which required more laborious work to prepare the adsorbent than the current adsorption preparation.

**TABLE 4:** Comparison of the proposed method with other methods for adsorption anthocyanin.

Adsorbent	Sample	Adsorption capacity (mg/g)	Reference
Oasis® MCX sorben Sep‐pak® C18 cartridge	Fruits and vegetables	45.8 19.0	He & Giusti et al. (2011)
Amberlite XAD7HP	Red cabbage	0.84 mg/mL	Chandrasekhar et al. (2012)
Porous clay	Purple Cabbage	16.89	Guimarães et al. (2023)
XAD7HP resin FPX66 resin XAD1180 resin	Blueberries	25.8 24.1 23.9	Buran et al. (2014)
Modified bentonites	Saffron tepals	1.55–2.25	This work

## 4 CONCLUSION

In this study, anthocyanin extract from saffron tepals was obtained using the extraction method utilizing ultrasonication. The adsorption of anthocyanin was done well by employing the adsorption procedure onto modified bentonite, which demonstrated higher adsorption capacities than raw bentonite. The adsorption process was determined through equilibrium isotherms, thermodynamics, and kinetics. Langmuir isotherm model was proposed for the adsorption of anthocyanin from saffron tepals. The investigation of adsorption thermodynamic analysis indicated that the adsorption process was spontaneous (negative quantity of ∆*G* ^0^) and exothermic with adsorption anthocyanin in the liquid–solid interface that entropy was decreased (Δ*S* ^0^). Adsorption data kinetics of anthocyanin were well‐matched with a pseudo‐second‐order kinetic model. The diffusion model indicated that the adsorption process was conducted through several diffusion mechanisms. Generally, an environmentally friendly method is used to adsorb anthocyanin molecules from saffron tepals with a valuable, simple, effective operating technology, and low cost.

## AUTHOR CONTRIBUTIONS

**Hasan Oliaei Torshizi:** Data curation (equal); formal analysis (equal); investigation (equal); methodology (equal); validation (equal); writing – review and editing (equal). **Samieh Oliaei:** Conceptualization (equal); data curation (equal); formal analysis (equal); investigation (equal); software (equal); supervision (equal); writing – review and editing (equal).

## CONFLICT OF INTEREST STATEMENT

The authors declare that there are no known competing personal relationships or financial interests that could have appeared to influence the work reported in this paper.

## Data Availability

Data used are available on request from the authors.

## References

[fsn34388-bib-0001] Abbasvali, M. , Ranaei, A. , Shekarforoush, S. S. , & Moshtaghi, H. (2016). The effects of aqueous and alcoholic saffron (*Crocus sativus*) tepal extracts on quality and shelf‐life of pacific white shrimp (*Litopeneous vannamei*) during iced storage. Journal of Food Quality, 39, 732–742. 10.1111/jfq.12225

[fsn34388-bib-0002] Alkan, M. , Demirbaş, Ö. , & Doğan, M. (2007). Adsorption kinetics and thermodynamics of an anionic dye onto sepiolite. Microporous and Mesoporous Materials, 101, 388–396. 10.1016/j.micromeso.2006.12.007

[fsn34388-bib-0003] Boyd, G. E. , Adamson, A. W. , & Myres, L. S. (1947). Kinetics of ionic exchange adsorption processes. Journal of the American Chemical Society, 69, 2836–2848. 10.1021/ja01203a066 20270838

[fsn34388-bib-0004] Brouillard, R. (1983). The in vivo expression of anthocyanin colour in plants. Phytochemistry, 22, 1311–1323. 10.1016/S0031-9422(00)84008-X

[fsn34388-bib-0005] Buran, T. J. , Sandhu, A. K. , Li, Z. , Rock, C. R. , Yang, W. W. , & Gu, L. (2014). Adsorption/desorption characteristics and separation of anthocyanins and polyphenols from blueberries using macroporous adsorbent resins. Journal of Food Engineering, 128, 167–173. 10.1016/j.jfoodeng.2013.12.029

[fsn34388-bib-0006] Castañeda‐Ovando, A. , de Lourdes Pacheco‐Hernández, M. , Páez‐Hernández, M. E. , Rodríguez, J. A. , & Galán‐Vidal, C. A. (2009). Chemical studies of anthocyanins: A review. Food Chemistry, 113, 859–871. 10.1016/j.foodchem.2008.09.001

[fsn34388-bib-0008] Chandrasekhar, J. , Madhusudhan, M. C. , & Raghavarao, K. S. M. S. (2012). Extraction of anthocyanins from red cabbage and purification using adsorption. Food and Bioproducts Processing, 90, 615–623. 10.1016/j.fbp.2012.07.004

[fsn34388-bib-0009] Chen, Y. , Zhang, W. , Zhao, T. , Li, F. , Zhang, M. , Li, J. , Zou, Y. , Wang, W. , Cobbina, S. J. , Wu, X. , & Yang, L. (2016). Adsorption properties of macroporous adsorbent resins for separation of anthocyanins from mulberry. Food Chemistry, 194, 712–722. 10.1016/j.foodchem.2015.08.084 26471611

[fsn34388-bib-0010] Das, A. B. , Goud, V. V. , & Das, C. (2018). Adsorption/desorption, diffusion, and thermodynamic properties of anthocyanin from purple rice bran extract on various adsorbents. Journal of Food Process Engineering, 41, e12834. 10.1111/jfpe.12834

[fsn34388-bib-0011] Deineka, V. I. , Doronin, A. G. , Oleinits, E. Y. , Blinova, I. P. , Deineka, L. A. , & Chulkov, A. N. (2020). Sorption of Anthocyanins on bentonite clay. Russian Journal of Physical Chemistry A, 94, 1224–1229. 10.1134/S0036024420060072

[fsn34388-bib-0012] Duarte Neto, J. F. , Pereira, I. D. S. , Da Silva, V. C. , Ferreira, H. C. , Neves, G. D. A. , & Menezes, R. R. (2018). Study of equilibrium and kinetic adsorption of rhodamine B onto purified bentonite clays. Cerâmica, 64, 598–607. 10.1590/0366-69132018643722429

[fsn34388-bib-0013] Emam, H. E. , & Abdelhameed, R. M. (2022). Separation of anthocyanin from roselle extract by cationic nano‐rode ZIF‐8 constructed using removable template. Journal of Molecular Structure, 1267, 133607. 10.1016/j.molstruc.2022.133607

[fsn34388-bib-0014] Francis, F. J. , & Markakis, P. C. (1989). Food colorants: Anthocyanins. Critical Reviews in Food Science & Nutrition, 28, 273–314. 10.1080/10408398909527503 2690857

[fsn34388-bib-0015] Fuleki, T. , & Francis, F. J. (1968). Quantitative methods for anthocyanins. 2. Determination of total anthocyanin and degradation index for cranberry juice. Journal of Food Science, 33, 78–83. 10.1111/j.1365-2621.1968.tb00888.x

[fsn34388-bib-0016] Gerçel, Ö. , Özcan, A. , Özcan, A. S. , & Gercel, H. F. (2007). Preparation of activated carbon from a renewable bio‐plant of Euphorbia rigida by H_2_SO_4_ activation and its adsorption behavior in aqueous solutions. Applied Surface Science, 253, 4843–4852. 10.1016/j.apsusc.2006.10.053

[fsn34388-bib-0017] Gonzalez‐Pradas, E. , Villafrance‐Sanchez, M. , Fernandez‐Perez, A. M. , & Socias Viciana, M. (1993). Adsorption of malathion from aqueous solution on homoionic bentonite samples. Agrochimica, 37, 104–110.

[fsn34388-bib-0018] Goupy, P. , Vian, M. A. , Chemat, F. , & Caris‐Veyrat, C. (2013). Identification and quantification of flavonols, anthocyanins and lutein diesters in tepals of *Crocus sativus* by ultra performance liquid chromatography coupled to diode array and ion trap mass spectrometry detections. Industrial Crops and Products, 44, 496–510. 10.1016/j.indcrop.2012.10.004

[fsn34388-bib-0019] Guimarães, D. T. , Mendes, L. M. R. , de Sousa Sabino, L. B. , de Brito, E. S. , Vilarrasa‐García, E. , Rodríguez‐Castellón, E. , Cecilia, J. A. , & da Silva Junior, I. J. (2023). Partial purification of anthocyanins (*Brassica oleracea* var. Rubra) from purple cabbage using natural and modified clays as adsorbent. Adsorption Science & Technology, 2023. 10.1155/2023/2724122

[fsn34388-bib-0020] He, J. , & Giusti, M. M. (2011). High‐purity isolation of anthocyanins mixtures from fruits and vegetables—A novel solid‐phase extraction method using mixed mode cation‐exchange chromatography. Journal of Chromatography A, 1218, 7914–7922. 10.1016/j.chroma.2011.09.005 21968344

[fsn34388-bib-0021] Hemati Kakhki, A. (2010). Stability of anthocyanin extracted from saffron (Crocus sativus L.) petals in a model beverage. Acta Horticulturae, 850, 247–250. 10.17660/ActaHortic.2010.850.42

[fsn34388-bib-0022] Ho, Y. S. , & McKay, G. (1998). A comparison of chemisorption kinetic models applied to pollutant removal on various sorbents. Process Safety and Environmental Protection, 76, 332–340. 10.1205/095758298529696

[fsn34388-bib-0023] Ikram, M. , Rauf, M. A. , & Rauf, N. (2002). Trace level removal studies of Cr (III) from aqueous solution. Journal of Trace and Microprobe Techniques, 20, 119–125. 10.1081/TMA-120002465

[fsn34388-bib-0024] Javed, S. H. , Zahir, A. , Khan, A. , Afzal, S. , & Mansha, M. (2018). Adsorption of Mordant Red 73 dye on acid activated bentonite: Kinetics and thermodynamic study. Journal of Molecular Liquids, 254, 398–405. 10.1016/j.molliq.2018.01.100

[fsn34388-bib-0025] Jiang, X. , Wang, M. , Lou, Z. , Han, H. , Yan, N. , Guan, Q. , & Xu, L. (2024). Selective and controlled release responsive nanoparticles with adsorption‐pairing synergy for anthocyanin extraction. ACS Nano, 18, 2290–2301. 10.1021/acsnano.3c10131 38207222

[fsn34388-bib-0026] Kooli, F. , Liu, Y. , Al‐Faze, R. , & Al Suhaimi, A. (2015). Effect of acid activation of Saudi local clay mineral on removal properties of basic blue 41 from an aqueous solution. Applied Clay Science, 116, 23–30. 10.1016/j.clay.2015.07.044

[fsn34388-bib-0027] Kozar, S. , Bilinski, H. , Branica, M. , & Schwuger, M. J. (1992). Adsorption of Cd (II) and Pb (II) on bentonite under estuarine and seawater conditions. Science of the Total Environment, 121, 203–216. 10.1016/0048-9697(92)90316-K

[fsn34388-bib-0028] Kubo, I. , & Kinst‐Hori, I. (1999). Flavonols from saffron flower: Tyrosinase inhibitory activity and inhibition mechanism. Journal of Agricultural and Food Chemistry, 47, 4121–4125. 10.1021/jf990201q 10552777

[fsn34388-bib-0029] Lian, L. , Guo, L. , & Guo, C. (2009). Adsorption of Congo red from aqueous solutions onto Ca‐bentonite. Journal of Hazardous Materials, 161, 126–131. 10.1016/j.jhazmat.2008.03.063 18487014

[fsn34388-bib-0030] Mahboub, R. , El Mouzdahir, Y. , Elmchaouri, A. , Carvalho, A. , Pinto, M. , & Pires, J. (2006). Characterization of a delaminated clay and pillared clays by adsorption of probe molecules. Colloids and Surfaces A: Physicochemical and Engineering Aspects, 280, 81–87. 10.1016/j.colsurfa.2006.01.036

[fsn34388-bib-0031] Mall, I. D. , Srivastava, V. C. , Agarwal, N. K. , & Mishra, I. M. (2005). Removal of Congo red from aqueous solution by bagasse fly ash and activated carbon: Kinetic study and equilibrium isotherm analyses. Chemosphere, 61, 492–501. 10.1016/j.chemosphere.2005.03.065 15869781

[fsn34388-bib-0032] Moshiri, E. , Basti, A. A. , Noorbala, A. A. , Jamshidi, A. H. , Abbasi, S. H. , & Akhondzadeh, S. (2006). Crocus sativus L.(petal) in the treatment of mild‐to‐moderate depression: A double‐blind, randomized and placebo‐controlled trial. Phytomedicine, 13, 607–611. 10.1016/j.phymed.2006.08.006 16979327

[fsn34388-bib-0033] Norman, M. , Bartczak, P. , Zdarta, J. , Ehrlich, H. , & Jesionowski, T. (2016). Anthocyanin dye conjugated with Hippospongia communis marine demosponge skeleton and its antiradical activity. Dyes and Pigments, 134, 541–552. 10.1016/j.dyepig.2016.08.019

[fsn34388-bib-0034] Olguin, M. T. , Solache‐Rios, M. , Acosta, D. , Bosch, P. , & Bulbulian, S. (1997). UO _2_ ^2+^ sorption on bentonite. Journal of Radioanalytical and Nuclear Chemistry, 218, 65–69. 10.1007/BF02033975

[fsn34388-bib-0035] Özcan, A. S. , Erdem, B. , & Özcan, A. (2005). Adsorption of Acid Blue 193 from aqueous solutions onto BTMA‐bentonite. Colloids and Surfaces A: Physicochemical and Engineering Aspects, 266, 73–81. 10.1016/j.colsurfa.2005.06.001

[fsn34388-bib-0036] Panda, A. K. , Mishra, B. G. , Mishra, D. K. , & Singh, R. K. (2010). Effect of sulphuric acid treatment on the physico‐chemical characteristics of kaolin clay. Colloids and Surfaces A: Physicochemical and Engineering Aspects, 363, 98–104. 10.1016/j.colsurfa.2010.04.022

[fsn34388-bib-0037] Rodriguez, M. V. , González, J. D. D. L. , & Munoz, M. B. (1995). Preparation of microporous solids by acid treatment of a saponite. Microporous Materials, 4, 251–264. 10.1016/0927-6513(95)00011-W

[fsn34388-bib-0038] Sánchez‐Vioque, R. , Rodríguez‐Conde, M. F. , Reina‐Ureña, J. V. , Escolano‐Tercero, M. A. , Herraiz‐Peñalver, D. , & Santana‐Méridas, O. (2012). In vitro antioxidant and metal chelating properties of corm, tepal and leaf from saffron (*Crocus sativus* L.). Industrial Crops and Products, 39, 149–153. 10.1016/j.indcrop.2012.02.028

[fsn34388-bib-0039] Sposito, G. (1984). The surface chemistry of soils. Oxford University Press.

[fsn34388-bib-0040] Taylor, R. K. (1985). Cation exchange in clays and mudrocks by methylene blue. Journal of Chemical Technology and Biotechnology. Chemical Technology, 35, 195–207. 10.1002/jctb.5040350407

[fsn34388-bib-0041] Teng, M. Y. , & Lin, S. H. (2006). Removal of basic dye from water onto pristine and HCl‐activated montmorillonite in fixed beds. Desalination, 194, 156–165. 10.1016/j.desal.2005.11.008

[fsn34388-bib-0042] Toor, M. , & Jin, B. (2012). Adsorption characteristics, isotherm, kinetics, and diffusion of modified natural bentonite for removing diazo dye. Chemical Engineering Journal, 187, 79–88. 10.1016/j.cej.2012.01.089

[fsn34388-bib-0043] Weber, W. J. , & Morris, J. C. (1962, September). Advances in water pollution research. In Proceedings of the First International Conference on water pollution research (Vol. 2, pp. 231–266). Pergamon Press Oxford.

[fsn34388-bib-0044] Xue, H. K. , Shen, L. Y. , Wang, X. R. , Liu, C. , Liu, C. , Liu, H. , & Zheng, X. (2019). Isolation and purification of anthocyanin from blueberry using macroporous resin combined Sephadex LH‐20 techniques. Food Science and Technology Research, 25, 29–38. 10.3136/fstr.25.29

[fsn34388-bib-0045] Xue, H. K. , Tan, J. Q. , Li, Q. , Tang, J. T. , & Cai, X. (2021). Counter‐current fractionation‐assisted and bioassay‐guided separation of active compounds from cranberry and their interaction with α‐glucosidase. LWT—Food Science and Technology, 145, 111374. 10.1016/j.lwt.2021.111374 PMC7998573 33804322

[fsn34388-bib-0046] Xue, H. K. , Zhu, X. H. , Tan, J. Q. , Fan, L. L. , Li, Q. , Tang, J. T. , & Cai, X. (2021). Counter‐current fractionation‐assisted bioassay‐guided separation of active compound from blueberry and the interaction between the active compound and α‐glucosidase. Food, 10, 509. 10.3390/foods10030509 PMC7998573 33804322

[fsn34388-bib-0047] Yoshida, K. , Kitahara, S. , Ito, D. , & Kondo, T. (2006). Ferric ions involved in the flower color development of the Himalayan blue poppy, *Meconopsis grandis* . Phytochemistry, 67, 992–998. 10.1016/j.phytochem.2006.03.013 16678868

